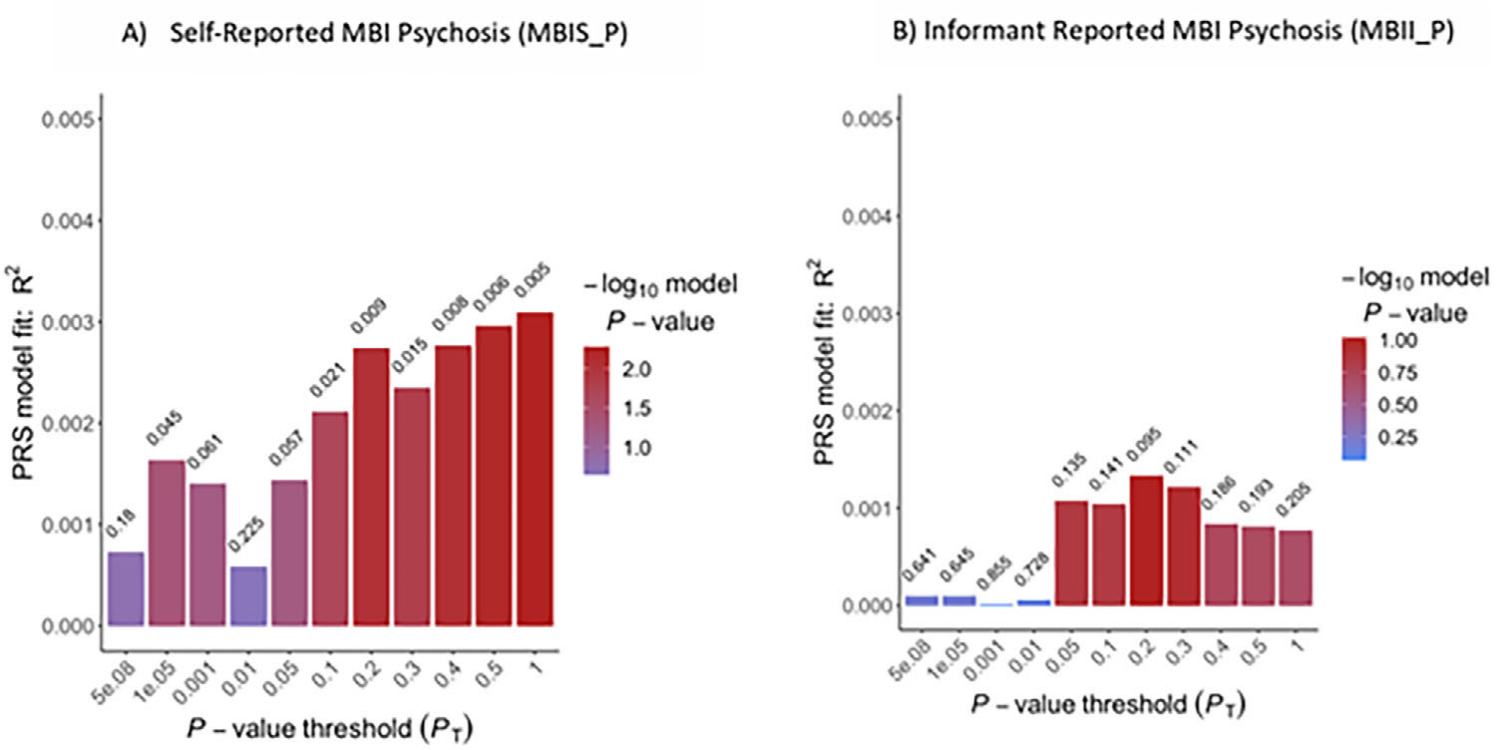# Examining the shared genetic liability between late‐life onset psychosis and major psychiatric, cognitive and personality phenotypes

**DOI:** 10.1002/alz.088683

**Published:** 2025-01-03

**Authors:** Byron Creese, Zahinoor Ismail, Clive G Ballard, Anne Corbett, Dag Aarsland, Olly Gibbs, Ellie Moodie

**Affiliations:** ^1^ Brunel University London, London United Kingdom; ^2^ Hotchkiss Brain Institute, University of Calgary, Calgary, AB Canada; ^3^ University of Exeter, Exeter, Devon United Kingdom; ^4^ College of Medicine and Health, University of Exeter, Exeter United Kingdom; ^5^ King’s College London, London, England United Kingdom; ^6^ University of Exeter, Exeter United Kingdom

## Abstract

**Background:**

When assessed in the Mild Behavioral Impairment (MBI) framework, late‐life onset psychotic like symptoms (MBI‐psychosis) are associated with incident cognitive decline and dementia. One approach to examining the genetic basis of this association, is to use Polygenic Risk Scores (PRS) to determine whether genetic propensity for late‐life onset psychosis is shared with other traits. We aimed to elucidate the shared genetic liability between Educational Attainment, Intelligence, Reasoning, Memory, Neuroticism, Alzheimer’s Disease, Major Depression, Schizophrenia and Bipolar Disorder and Mild Behavioral Impairment (MBI)‐Psychosis in later life.

**Method:**

A total of 7,307 older adults without dementia were included in the analytical sample. MBI‐Psychosis status (present or absent) was determined by the Mild Behavioral Impairment Checklist (MBI‐C) rated by participants and study partners (that is ‘self’ and ‘informant’ ratings). Each PRS was tested in a logistic regression model with MBI‐Psychosis status as the dependent variable, and with age, sex and ancestry as covariates.

**Result:**

Higher PRS for Major Depression, Schizophrenia and Neuroticism were all associated with an higher odds of MBI‐Psychosis. PRS for schizophrenia was only associated with self‐reported MBI‐psychosis, not informant reported MBI‐psychosis (see Figure 1). Higher PRS for Educational Attainment and Intelligence were both associated with lower odds of MBI‐Psychosis. In analysis stratified by self‐reported education level, the relationship between higher PRS for educational attainment and lower odds of MBI‐psychosis was only present in those we left school at 16.

**Conclusion:**

In early life, psychosis is known to overall with cognitive, psychiatric and personality traits. These data extend this observation to later‐life psychosis. The significance of the differences between self and informant reported symptoms are yet to be determined but may be a mix of measurement error and the different respondents having a propensity to report symptoms which reflect different etiologies. We also show that established protective factors against cognitive decline, like educational attainment, in later life may also extend to late life neuropsychiatric syndrome.